# Successful Biological Treatment of a Patient With Psoriasis and HIV

**DOI:** 10.7759/cureus.71970

**Published:** 2024-10-20

**Authors:** Angel E Ortegon Blanco, Maria E Alonzo Canul

**Affiliations:** 1 Dermatology, Centro Medico Nacional 20 de Noviembre, Mexico City, MEX; 2 Dermatology, Instituto De Seguridad Y Servicios Sociales de Los Trabajadores Del Estado Merida Susula, Merida, MEX

**Keywords:** biological therapy, clinical case report, controlled hiv, hiv and psoriasis, psoriasis management

## Abstract

The use of biological agents for the management of chronic dermatological pathologies such as psoriasis is becoming more common every day. Until now, antibodies against tumor necrosis factors (TNF-α) inhibitors, interleukin IL-17 inhibitors, IL-12/23 inhibitors, and IL-23 inhibitors have been marketed as biological therapies for psoriasis, showing excellent results with a minimal number of adverse events. Initially, the first biological treatments were associated primarily with opportunistic infections. Later, specific adverse events were described depending on the biological agent used: TNF-α inhibitors were linked to tuberculosis, while anti-IL-17 agents were associated with candidiasis and worsening of inflammatory bowel disease (IBD). However, such therapies warrant a cautious screening protocol to detect pre-existing infections, mainly tuberculosis and chronic viral infections such as hepatitis. Human immunodeficiency virus (HIV) is only screened in case of suspicion and in some institutional protocols. We present the clinical case of a person living with HIV under optimal virological control, in which the use of biological therapy was imperative to control psoriasis and he also presented with great severity. Until now there is no official statement on the use or not of biologicals in this particular population.

## Introduction

Psoriasis is a systemic inflammatory disorder with a chronic component caused by an overactive immune system, primarily affecting the skin and joints. It has multiple clinical presentations, but the most common is plaque psoriasis, also known as psoriasis vulgaris, which manifests as erythematous-squamous lesions mostly located on the scalp, trunk, and extensor surfaces of the limbs, although other regions may also be involved. These lesions often cause itching of variable intensity [1]. It affects between 0.9% and 5.1% of the global population; however, epidemiological figures for psoriasis are very limited (described in only 20 countries). Therefore, it is estimated that the actual prevalence ranges from 0.51% to 11.43% of the adult population and from 0% to 1.37% in children [2]. Up to date, the diagnosis is based on clinical characteristics with the add-up of skin lesions, their morphology, location, and scaling state. Usually, a skin biopsy is performed to exclude other conditions or to confirm the suspected diagnosis [3]. Depending on the clinical severity, extent, and type of lesion, as well as the patient's preferences and comorbidities, the treatment will be determined. The goals of the treatment in psoriasis mainly consist of reducing inflammation, eliminating skin lesions, improving quality of life, and preventing complications. Topical corticosteroids, keratolytics, vitamin D analogs, and topical calcineurin inhibitors are the conventional first-line therapy for mild psoriasis [4]. In the last 15 years, advances in understanding the pathogenesis of psoriasis have translated into targeted and highly effective therapies using biological treatments, the main ones with real-world clinical experience being antibodies against tumor necrosis factor-alpha (TNF-α), and interleukins (IL) 12, 17, 21, and 23 [5].

Biological therapy refers to the use of molecules, particularly antibodies, derived from human cells or another living organism, which have a highly specific action on certain molecular signaling pathways and/or cellular receptors, with the aim of stimulating, inhibiting, or modulating the immune system and inflammatory reactions [6]. Due to alterations in immune response, patients receiving these agents are particularly susceptible to severe infections caused by opportunistic microorganisms (odds ratio (OR): 1.37, 95% CI: 1.04-1.82), especially viruses (e.g., herpes), fungi (e.g., Histoplasma capsulatum), and mycobacteria, with a high risk of tuberculosis reactivation in the latter case (OR: 4.68, 95% CI: 1.18-18.60). Since tuberculosis is endemic in Mexico, it is recommended to rule out active or latent infections before starting any biological therapy [7].

The following is a case presentation of a patient with plaque psoriasis. Given the severity of the dermatological lesions and lack of response to other systemic therapies, it was decided to initiate treatment with biological therapy, despite the patient's long-standing medical history of human immunodeficiency virus (HIV) infection under optimal virological control.

## Case presentation

The case involves a 28-year-old male native and resident of Mexico. The patient's significant medical history includes a diagnosis of HIV infection in February 2015. He began antiretroviral therapy (ART) and achieved undetectable viral load levels within three months that same year, which he has maintained in all subsequent follow-ups. His latest check-up in May 2023 showed an undetectable viral load and a CD4 lymphocyte count of 679 cells/mm³. He is currently receiving bictegravir/tenofovir alafenamide/emtricitabine every 24 hours as ART.

**Figure 1:**
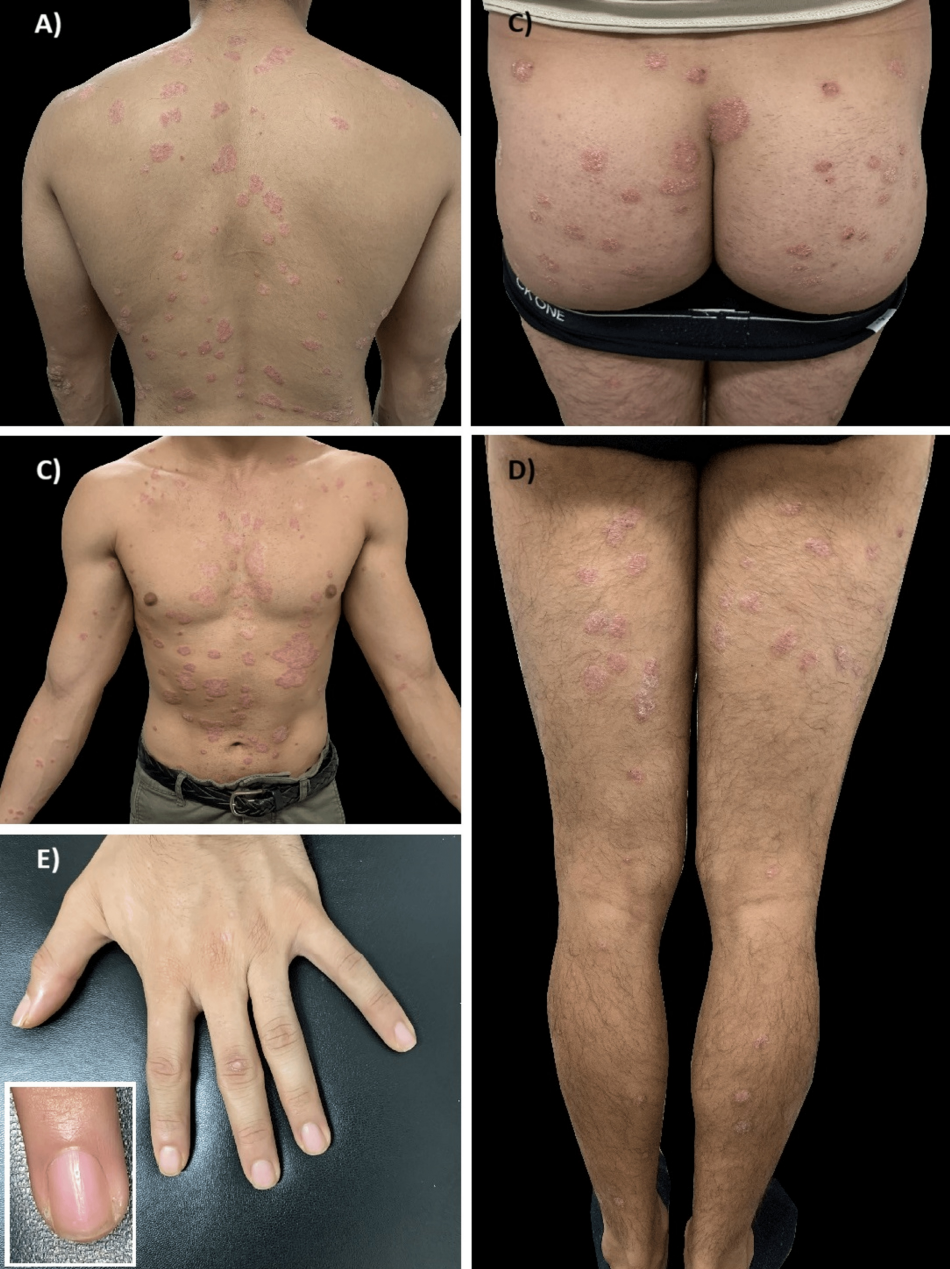
Psoriasis plaques: A) posterior trunk, B) anterior trunk and abdomen, C) glutes, D) legs, and E) hands

In 2017, the patient developed a disseminated dermatosis on his head, neck, chest, and upper and lower limbs, characterized by multiple irregular, well-defined erythematous-squamous plaques. He was clinically diagnosed with psoriasis, with a Psoriasis Area and Severity Index (PASI) of 10.3. He was treated with methotrexate; however, the treatment was interrupted in 2020 due to a lack of continuity in non-priority health services caused by the COVID-19 pandemic. He returned to the dermatology clinic in February 2022, presenting with psoriasis plaques on his back, chest, abdomen, buttocks, hands, and lower limbs (Figure 1).

Oral methotrexate was reinitiated, along with topical treatment using coal tar at night and 20% urea in the mornings. Due to a poor response to the treatment, the methotrexate dose was increased to 17.5 mg on Saturdays, but there was still no satisfactory response. Consequently, in February 2023, it was decided to start treatment with Secukinumab (300 mg subcutaneously monthly) and gradually reduce methotrexate.

**Figure 2:**
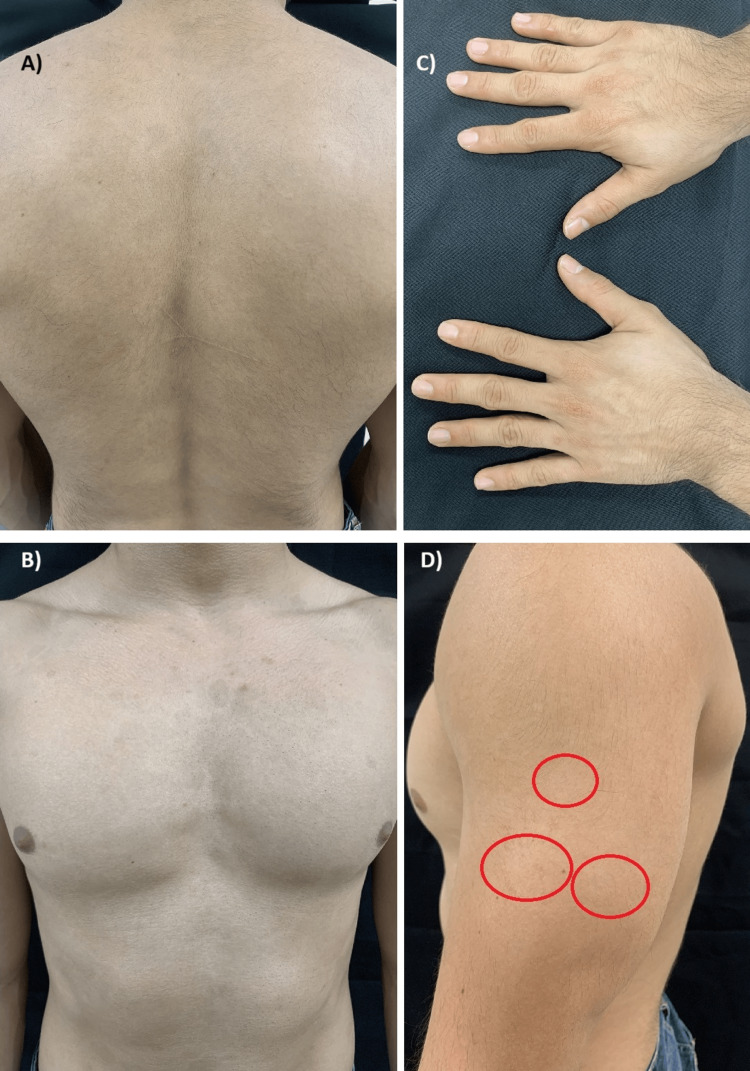
Resolution of psoriasis plaques: A) posterior trunk, B) anterior trunk and abdomen, C) hands, and D) small persistent desquamative zones on the left arm

The patient experienced a reduction in itching and scaling from the third day after receiving the biological treatment. After ten months of treatment, some hyper- and hypopigmented post-inflammatory macules and fine scales persisted on his head and left arm (Figure 2), resulting in a PASI of 0.1. No abnormalities were documented in blood chemistry, blood count, HIV viral load, or CD4 lymphocyte count during follow-up.

## Discussion

It is the complex interaction of genetics, environment, and dysregulation of the immune system, both innate and adaptive, that results in psoriasis. An inflammatory cascade that starts with the activation of the innate immune system and keeps involving the release of pro-inflammatory cytokines, migration of antigen-presenting cells, and finally the activation and differentiation of T lymphocytes into polarized T cells mostly Th1, Th17, and Th22 [8]. The upregulation of these polarized T cells cytokines (i.e., IFN-ɣ, TNF-α, IL-17A, IL-17F, and IL-22) perpetuates a chronic inflammation process that leads to abnormal proliferation and differentiation of epidermal keratinocytes [9]. "The likely positive response to biological treatment in this patient may be related to his non-detectable viral load and CD4 count of over 500 cells/mm³ throughout the follow-up. Additionally, adopting healthy lifestyle habits, such as daily exercise and a balanced diet, helps reduce metabolic risks.

Secukinumab is a recombinant human monoclonal antibody that neutralizes IL-17A by selectively binding to it. It was approved by the United States Food and Drug Administration (FDA) in 2015, followed by ixekizumab, approved in 2016, and later by brodalumab (IL-17RA), and guselkumab (IL-23) in 2017. Secukinumab has a weekly dose of 300 mg for the first five weeks, followed by 300 mg every four weeks, and is administered by subcutaneous injection [10].

In the presented case, our patient had used a disease-modifying drug with a poor response as a baseline treatment for moderate psoriasis by PASI. Once biological treatment was started a very rapid response was experienced within just three days, which was maintained throughout the follow-up. Regarding this, secukinumab showed a rapid onset of action, significantly likely to achieve PASI 75 within the first week of treatment compared to ustekinumab, with clinical superiority by week 16 [11]. After 12 weeks of receiving secukinumab, 81.6% of patients achieved a PASI 75 response, and 28.6% achieved a PASI 100 response. By week 52, over 80% maintained PASI 75. Our patient had been on follow-up for 40 weeks with an achieved PASI 90 response that corresponds to the described literature.

There are two key clinical studies, ERASURE and FIXTURE, with results of PASI 75 achieved by week 12 in 77.1%, and 81.6% of patients who received 300 mg of secukinumab, and 67.0% and 71.6% of patients who received 150 mg of secukinumab; notably, with better clinical effects compared to etanercept at a 50 mg dose [12]. A Controlled clinical trial done in 2019 of patients receiving secukinumab versus placebo found that suppression of the IL-23/IL-17 axis by secukinumab was evident by week one and continued until week 12, which was associated with clinical and histological responses, confirming that secukinumab can reverse changes caused by hyperplastic keratinocytes [13].

Historically, when psoriasis and HIV-positive are concomitants in a patient it usually tends to have a more severe clinical manifestation, faster progression, and is usually resistant to first and second-line therapies, this includes topical therapy, phototherapy, and systemic retinoids. Our patient had a poor response to the systemic agent methotrexate in combination with topical agents. Another systemic agent commonly used on psoriasis is cyclosporine, both with their respective immunosuppressive effects, which are the main reason some dermatologists don't feel comfortable using them in HIV-positive patients who already have an immunocompromised immunologic response [14]. Thus, until just a decade ago, the consensus among most dermatological societies was to use topical therapy as the first line for mild to moderate psoriasis and phototherapy for moderate to severe cases, with oral retinoids as the second line; and only for refractory or severe cases consider the cautious use of cyclosporine, methotrexate, hydroxyurea, and biologicals [15].

There is a paradoxical pathogenesis when psoriasis and HIV-infected patients are associated, and this is explained by imbalanced immune cell components. T cells mediate the excessive inflammatory response in psoriasis, while HIV infection depletes T lymphocytes (creating susceptibility to opportunistic infections) [16]. Additionally, psoriasis may worsen or be detected for the first time when HIV infection is diagnosed, further affecting the quality of life of HIV patients, or in already diagnosed HIV patients, psoriasis may appear later, in which case there seems to be a correlation between HIV virological control and psoriasis worsening [17].

However, biological drug safety and efficacy profiles in HIV-positive patients have not been well established due to exclusion from clinical trials of this patient population. Therefore, in 2023, a systematic review was conducted on this topic, finding only 100 studies that narrated the use of biologicals in 179 HIV-positive individuals. A favorable safety profile with minimal or minor adverse events has been documented in almost all classes of biological drugs; opportunistic infections are associated with anti-CD-20 inhibitors and TNF-α inhibitors, and there has been a transitory increase in HIV viral load with some biological agents like TNF-alpha inhibitors. The evidence on this matter is of low quality and is limited to case reports and retrospective reviews [18].

Gong et al. reported a case of an HIV-positive person on ART who presented with psoriasis vulgaris. As first-line treatment, the patient received oral acitretin and subcutaneous etanercept, but two months later, the dermatological condition worsened, leading to discontinuation and replacement with secukinumab. A rapid response was observed (within four weeks), with times to achieve PASI 40 at two weeks, PASI 75 at four weeks, PASI 90 at eight weeks, and PASI 100 at week 29. For one year, the treatment was maintained without adverse reactions. Viral load and CD4+ T lymphocyte count remained stable without alteration, determining that administrating anti-IL-17 monoclonal antibodies can be used as an effective treatment option in this population [19]. Therefore, in the present case, secukinumab was chosen as the first biological therapy with an equally successful outcome.

The use of biologicals is not free from adverse reactions. A recent 2021 non-systematic review compiled available information from 127 studies containing 1023 plaque psoriasis cases that received TNF-α, IL-12/23, IL-17, and IL-23 inhibitors, observing adverse event rates of 4.17%, 9.49%, 12.4%, and 7.37%, respectively. Specifically, for IL-17 inhibitors, skin infections were the most common (5.01%, n=512), with secukinumab presenting the lowest rate of this complication (2.93%, n=93). Other common complications within this group were local injection site reactions (4.68%, n=475), though minimal for specific cases of bimekizumab (0.3%, n=3) and Secukinumab (0.35%, n=11). Most adverse events are transient and rarely lead to discontinuation of the biological, as evidenced by a retrospective multicenter study (2005 to 2014) in Canada, analyzing 398 psoriasis patients on biologicals, with 22 cases (4.04%) presenting severe adverse events leading to cessation of the biological [20]. During the entire follow-up, the patient did not experience any adverse effects. It is important to note that the time the patient suspended treatment was not voluntary; it was due to modifications in public health services caused by the pandemic emergency.

This case report was prepared as a review of the patient during the clinical resident rotation. Throughout the follow-up, our patient did not present any adverse events and continues to have ongoing follow-up care.

## Conclusions

People living with HIV who adhere to ART are able to maximally suppress viral replication, resulting in their immune system no longer being significantly compromised. Therefore, in theory, the same protocols for the use of biologics could be applied, with very close monitoring and surveillance of virological control. However, the evidence is still scarce, which is why we decided to share this successful case, hoping that in the near future, more controlled studies will be conducted in our field to provide more information and generate greater evidence regarding the use of new biologics in patients with associated diseases.
